# Acceptability of mental health stigma-reduction training and initial effects on awareness among military personnel

**DOI:** 10.1186/s40064-015-1402-z

**Published:** 2015-10-13

**Authors:** Suzanne L. Hurtado, Cynthia M. Simon-Arndt, Jennifer McAnany, Jenny A. Crain

**Affiliations:** Health and Behavioral Sciences, Naval Health Research Center, 140 Sylvester Road, San Diego, CA 92106-3521 USA

**Keywords:** Stigma, Mental health, Military, Toolkit, Acceptability, Awareness

## Abstract

The purpose of this paper is to report on the development of a mental health stigma reduction toolkit and training, and the acceptability and level of stigma awareness following the stigma-reduction training for military personnel. The overall aims of the training were to provide discussion tools highlighting the experiences of Marines seeking help for stress concerns, improve communication between leaders and their Marines around the issue of help seeking, and familiarize Marines with behavioral health treatment. Senior enlisted leaders and officers (*N* = 52) from a Marine Corps battalion participated in a pretest, 2-h stigma-reduction training and immediate posttest. Acceptability of the training was measured by querying participants about the usefulness and helpfulness of the training among other factors, and stigma awareness was measured with 10 items about mental health stigma. The stigma-reduction training and materials were well accepted by participants. In addition, there was a significant improvement in four of ten stigma-reduction awareness concepts measured before and immediately after the training, which included an increase in agreement that mental health treatments are usually effective in reducing stress reactions [*t*(51) = −3.35, *p* = 0.002], and an increase in disagreement that seeking counseling after a deployment will jeopardize future deployments [*t*(51) = −3.05, *p* = 0.004]. Level of agreement with several statements including those regarding perceptions of invincibility, and malingering, among others, did not change significantly after the training. The stigma-reduction training containing educational and contact strategies was highly acceptable to the leaders and may have promise for initially dispelling myths associated with seeking help for stress concerns among military service members; however, results indicate that there is clearly more work to be done in combatting stigma.

## Background

Emerging from an era of high operational tempo and rotating deployments, military personnel are still experiencing high levels of psychological concerns including posttraumatic stress symptoms, anxiety disorders, depression and substance abuse (Bray et al. 2010; Hoge et al. 2004; Jacobson et al. 2008; Milliken et al. 2007). Unfortunately, many service members do not seek behavioral health treatment at all, and the stigma associated with seeking help for stress concerns may be preventing individuals from getting early treatment (Hoge et al. 2004). In particular, negative attitudes toward mental health treatment are associated with help-seeking intentions, as well as decreased utilization of mental health care in the military (Brown et al. 2011; Kim et al. 2011; Sharp et al. 2015).

There has been some evidence of reductions in stigma and negative attitudes in recent years (Quartana et al. 2014; Office of the Surgeon General, United States Army Medical Command, Office of the Command Surgeon HQ, USCENTCOM, and Office of the Command Surgeon US Forces Afghanistan (USFOR-A) 2011; Momen et al. 2012). These reductions may be due in part to anti-stigma efforts initiated in the military to address this concern (Acosta et al. 2014), including campaigns such as the Defense Centers of Excellence Real Warriors campaign and the web-based Afterdeploymnet.org. Service-specific efforts, such as those in the Marine Corps, raise awareness about the signs of combat and operational stress and aim to change negative cultural perceptions about help seeking. However, many of these programs have not been evaluated for their acceptability or impact on reducing stigma.

Moreover, despite recent improvements, a substantial proportion of personnel still endorse items reflecting stigmatizing beliefs. In the present paper, we report on the development of a stigma reduction toolkit and training designed to augment current efforts by directly counteracting negative beliefs associated with seeking care. We also present the acceptability of the stigma-reduction training and the initial effect on stigma awareness among a sample of military personnel.

## Methods

### Training

The program consisted of a 2-h training session with a companion toolkit for Marine Corps senior enlisted leaders (SELs), defined as those in E6–E9 pay grades, and officers to reduce the stigma of seeking help for stress concerns among their Marines. Both the content and approach of the toolkit were developed based on formative research conducted for this study, consultation with military mental health care providers, and interviews with active duty Marines and former military personnel. The materials were targeted to the SELs and officers because they (particularly the SELs) are the role models for junior Marines and set the tone for acceptable practices and attitudes in the unit. The overall aims of the training were to provide discussion tools highlighting the experiences of Marines seeking help for stress concerns, improve communication between leaders and their Marines around the issue of help seeking, and familiarize Marines with behavioral health treatment.

The primary approach utilized in the training was to increase the contact that participants have with military members who have experienced a stress injury. Corrigan and Penn (1999) explain that stigma tends to diminish when people have contact, either directly or indirectly (Reinke et al. 2004), with someone who has a mental health condition. Military researchers agree that a stigma-reduction approach incorporating increased contact with service members who have had these types of experiences is likely to increase acceptance of the messages and may enhance effectiveness (Greene-Shortridge et al. 2007). The toolkit in the present study highlighted the personal experiences of seven Marines: four of them were presented in written scenarios with short video clips or links and each included a discussion guide that a leader could use to engage their Marines in a discussion and encourage help seeking. Three additional article and video links of Marine’s personal stories with seeking help were included for which leaders could design and tailor their own discussions, and additional scenarios for discussion were also included. This form of indirect contact was structured to personalize and normalize the experiences highlighted in the stories for the Marines exposed to the training. Combining contact and educational strategies has been recommended for combatting stigma among military personnel (Ben-Zeev et al. 2012). Thus, the contact strategy in the present program was combined with educational information in several areas: behavioral health treatment, communication style for talking about stress concerns, resources for getting help and assessing stigma (Table 1). The stigma assessment was a checklist of factors, conditions and interactions in the work environment that may be affecting the level of stigma in the unit. The checklist included specific recommendations for improving the area(s) that the leader identified as needing attention.

**Table 1:** Content of the stigma reduction training and toolkit

Section	Main points
Discussion tools—personal experiences of Marines addressing stress injuries and other scenarios	Expected stress reactions Overcoming common reasons for not seeking help such as being perceived as weak, concerns about career consequences, concerns about security clearance, wanting to deal with problems on their own Seeking help early Recognizing opportunities to help a fellow Marine Support during recovery and reintegration
Behavioral health treatment	Benefits of getting help Barriers to getting help Confidentiality Continuum of care Types of providers Nature of treatment and common treatments Medications Communicating with providers Supporting a Marine in treatment
Effective communication style	Interactive discussion Active listening Tips for starting the conversation about getting help Increasing unit bonding and cohesion
Resources	Military and civilian resources for help and information
Assessing stigma	Checklist of conditions and actions that affect stigma in the work environment and suggestions for improvements

The toolkit familiarized Marines with behavioral health treatment and addressed mental health-related beliefs as suggested in two reviews of empirical research on barriers to care and stigma among military personnel (Vogt 2011; Dickstein et al. 2010). This section covered several aspects of seeking help such as defining the continuum of care, types of resources and providers, common treatment options and medications. A mental health provider video was included in the toolkit, which tackled issues related to beliefs about treatment that may serve as barriers to care (Sayer et al. 2009). This video depicted three different mental health professionals (one civilian former military and two active duty) responding to a variety of issues related to seeking care. It was structured so that it could be used in its entirety or in brief segments by topic area. Additionally, incorporated throughout the program was the ethos of “Marines take care of Marines” and repositioning the cultural conceptualization of strength in alignment with help seeking to empower Marines in the manner suggested by Dickstein et al. (2010).

The training was co-taught by an active duty Marine Corps Sergeant Major and a Veterans Affairs clinical psychologist. The Sergeant Major was selected as his rank and deployment experience would likely provide him credibility with the participants about the topic. Similarly, the clinical psychologist, who was actively treating patients with combat and operational stress concerns, was chosen to provide credibility when talking about issues such as the types of care, treatment effectiveness and confidentiality. The trainers briefed on the contents of the toolkit and showed how to use it by demonstrating, for example, how to talk one-to-one about a stress concern and how to conduct a group discussion about confidentiality and treatment options based on a segment from the mental health provider video.

### Participants

Participants were 52 active duty leaders, which included senior enlisted personnel (rank E6–E9) and officers from a Marine Corps infantry battalion. All participants were male as only males were assigned to the Marine Corps infantry battalion. A total of 57 service members initially participated in the pretest; however, five subjects did not participate in the posttest, yielding a 91 % posttest participation rate. The sample consisted of Marines with 52 % in the pay grades of E6–E9 and 48 % officers. Specifically, the sample contained the following breakdown of participants by rank: E6 (13), E7 (7), E8 (3), E9 (1), non-specified enlisted (3), W2 (1), O1 (4), O2 (11), O3 (5), O5 (1), and non-specific officers (3). The racial breakdown of participants was 83 % White, 8 % Hispanic/Latino, 5 % Black, 2 % Asian/Pacific Islander and 2 % other races/ethnicities. The majority of the sample was married (64 %) and had a bachelor’s degree or higher (48 %). The mean age was 30.9 years (*SD* = 5.3). The mean length of service was 10.3 years (*SD* = 6.15), with enlisted members having served more years on average (13.8) compared to officers (6.7). The majority of the sample (83 %) had been previously deployed.

### Procedures

Participants were recruited in summer 2012 by conducting an informed consent discussion with available members of the battalion. Consenting volunteers were administered a pretest, and then a posttest immediately after receiving a single training session on reducing stigma. All procedures for this study were approved by Naval Health Research Center’s Institutional Review Board.

### Measures

The measures focused on two aspects: acceptability of the training and awareness of stigma-reduction principles. Acceptability of the training was measured immediately after the training with seven individual items developed by the authors. These items queried participants about the usefulness and helpfulness of the training and the toolkit, as well as the toolkit’s ease of use and relevance, and their intentions to use the toolkit. Awareness of stigma-reduction principles was measured at both pretest and posttest with 10 items developed by the authors to assess specific concepts addressed in the training. These included statements about the nature of stress symptoms, seeking help, treatment and responsibility to assist others. Participants rated all acceptability and awareness items on a 5-point Likert-type scale (1 = strongly disagree, 5 = strongly agree).

### Data analysis

Descriptive statistics were used to examine acceptance of the training. Paired *t*-tests were conducted to examine differences in awareness from pretest to posttest. An alpha level of 5 % was assumed in all statistical tests. The analyses were conducted using IBM SPSS Statistics 19.0 (SPSS Inc.).

## Results

### Acceptability

Overall, 87 % of the 52 leaders who participated in the immediate posttest agreed or strongly agreed that the training was useful (Table 2). When specifically asked if the training helped them understand how reducing stigma will help their Marines, 52 % agreed and 25 % strongly agreed that it was helpful. Additionally, 92 % of leaders agreed or strongly agreed that the toolkit was useful for reducing stigma associated with seeking care for a stress injury, and nearly all Marines surveyed felt that the tools demonstrated in the training would be helpful for advising Marines about seeking care. Furthermore, 63 % of leaders agreed and 31 % strongly agreed that the tools were relevant to maintaining readiness, and approximately 92 % of participants agreed or strongly agreed that the materials were easy to use and that they intended to use them with their Marines. There were no significant differences in the acceptability measures between the enlisted leaders and the officers.

**Table 2:** Participants’ ratings of the training and toolkit

Item	Strongly disagree/disagree^a^ (%)	Neither agree nor disagree (%)	Agree (%)	Strongly agree (%)	Mean	*SD*
Training was useful	–	13.5	63.5	23.1	4.10	0.60
Training was helpful for understanding how reducing stigma will help my Marines	5.8	17.3	51.9	25.0	3.96	0.82
Toolkit was useful for reducing stigma	1.9	5.8	67.3	25.0	4.15	0.61
Toolkit was helpful for advising my Marines about seeking care	–	1.9	65.4	32.7	4.31	0.51
Toolkit was easy to use	1.9	5.8	59.6	32.7	4.23	0.64
Toolkit was relevant to maintaining readiness	–	5.8	63.5	30.8	4.25	0.56
I intend to use the toolkit	–	7.7	57.7	34.6	4.27	0.60

*N* = 52

Scale was 1 = ”Strongly disagree”, 2 = “Disagree”, 3 = “Neither agree nor disagree”, 4 = “Agree”, and 5 = “Strongly agree”

*SD* standard deviation

^a^Responses of “strongly disagree” and “disagree” were combined

### Stigma-reduction awareness

As shown in Table 3, four of the ten stigma-reduction awareness items showed a significant improvement from pretest to posttest. For example, leaders more strongly disagreed with the statement, “If you seek counseling after a deployment, you will not be able to deploy again” at the posttest immediately following the training than at pretest [*t*(51) = −3.05, *p* = 0.004]. In addition, leaders more strongly disagreed that “Only people with PTSD need to seek help” and “Mental health treatment always involves the use of medications” at the posttest than at pretest [*t*(51) = −3.32, *p* = 0.002 and *t*(51) = −3.44, *p* = 0.001, respectively]. Furthermore, there was a significant increase in agreement with the statement that mental health treatments are usually effective in reducing stress reactions [*t*(51) = −3.35, *p* = 0.002]. Level of agreement with several statements did not change significantly after the training, including statements such as, “Real Marines are immune to stress injuries,” and “Anyone who complains of stress reactions more than a day or two after a stressful situation is malingering,” among others.

**Table 3:** Participants’ stigma-reduction awareness at pretest and posttest

Item	Pretest		Posttest		*t*	*p*
	Mean	*SD*	Mean	*SD*		
If you seek counseling after a deployment, you will not be able to deploy again^a^	4.23	0.83	4.62	0.53	−3.05	0.004
Only people with PTSD need to seek help for their stress reactions^a^	4.31	0.83	4.67	0.51	−3.32	0.002
Mental health treatment always involves the use of medications^a^	4.12	0.94	4.52	0.70	−3.44	0.001
Real Marines are immune to stress injuries^a^	4.73	0.49	4.67	0.47	0.65	0.518
It is a leader’s responsibility to create an environment where it is OK to get help for a stress injury	4.37	0.91	4.54	0.73	−1.29	0.201
Mental health treatments are usually effective in reducing stress reactions	3.42	0.85	3.83	0.76	−3.35	0.002
Anyone who complains of stress reactions more than a day or two after a stressful situation is malingering^a^	4.20	0.85	4.40	0.77	−1.49	0.142
Negative comments about Marines who seek help for stress concerns are harmless^a^	4.33	0.86	4.52	0.70	−1.46	0.151
Getting help early for a stress concern should not damage a Marine’s career; however, negative consequences from waiting too long to seek help can damage a Marine’s career	3.80	1.17	4.06	1.03	−1.52	0.109
It is important for leaders to talk to Marines with stress concerns and encourage them to seek help	4.54	0.58	4.54	0.50	0.00	1.00

*N* = 52

Scale was 1 = ”Strongly disagree”, 2 = “Disagree”, 3 = “Neither agree nor disagree”, 4 = “Agree”, and 5 = “Strongly agree”

*SD* standard deviation, *PTSD* posttraumatic stress disorder

^a^Scale was reverse coded as 1 = ”Strongly agree”, 2 = “Agree”, 3 = “Neither agree nor disagree”, 4 = “Disagree”, and 5 = “Strongly disagree”

Because the sample of leaders was made up of enlisted personnel and officers who differed in time in service and may have held different points of view with regard to behavioral health issues, we conducted a post hoc analysis on changes in stigma-related awareness for the enlisted leaders and officers, separately. This analysis showed that the officer group had significant positive changes (*p* ≤ 0.01) in the same four items as when the whole sample was used, plus one additional item for which officers were more likely to agree that getting help early is better regarding concerns about career damage [*t*(23) = −2.50, *p* = 0.020]. The enlisted group did not show significant changes in any of the ten awareness items.

## Discussion

The results provide preliminary support for the acceptance of the stigma-reduction training and materials among the military leaders. A majority of the participants agreed that the training and materials would be helpful for advising Marines about seeking care. In addition, the immediate improvement in four of ten stigma-reduction awareness concepts among the leaders overall is positive. In particular, the increased endorsement of positive beliefs about the efficacy of mental health treatment is an important finding. Other military research (Kim et al. 2011) has found that negative attitudes about mental health treatment inversely predict treatment seeking. Our findings from the present study suggest that exposure to a briefing from a practitioner can increase confidence that seeking professional help for a mental health concern will be beneficial. This is encouraging because if Marine leaders think that treatments are going to be helpful, they will be more likely to support their members getting assistance and communicate the view that help-seeking is acceptable. While positive ratings of the materials and increased awareness alone do not translate to stigma-reducing actions, they are an important first step.

At the same time, however, there was no significant change in several of the other stigma awareness items. These items addressed issues of perceived invincibility, malingering, career concerns, and responsibility to encourage help seeking in various ways. One possible explanation for why the training appeared to have had more of an impact on the participants’ perspectives on treatment-related issues (i.e., counseling and redeployment; medications, treatment effectiveness) and less of an impact on nontreatment-related stigma issues is that the training was delivered, in part, by a clinician. Additionally, the video segments in the toolkit may have made the clinical aspects more salient. Another possibility is that some of the stigma awareness statements related to career concerns and perceived weakness may represent more strongly held beliefs that are more difficult to change than others. In addition, when officers and senior enlisted leaders were analyzed separately, only the officers showed significant improvements in stigma awareness; the enlisted leaders did not. Senior enlisted leaders may represent a group that has more strongly held beliefs and may need more intense or sustained efforts to impact their stigma-related attitudes.

The primary limitation in this study was the one-group pretest–posttest design for the assessment of change in stigma awareness. The results should be interpreted with caution due to the threats to internal validity inherent in this design. Using this design, other explanations for the observed improvements in stigma awareness cannot be ruled out. In addition, measurement-specific threats to internal validity may exist. The investigator-developed stigma awareness items are somewhat limiting and the use of a previously validated stigma measure would have strengthened this study. Also, the use of self-reported data is a limitation inherent in the nature of measuring concepts such as awareness. Participants may misreport for a variety of reasons to include social desirability among other factors.

## Conclusion

This study showed that the stigma-reduction training had overall high acceptability among the target audience and there was an immediate improvement in a small number of stigma awareness concepts post training. Given that the measured improvements in stigma awareness were somewhat minimal, the primary benefit of this program may be in providing leaders with tools to engage their service personnel about the importance of seeking help. The acceptability ratings indicated that nearly all of the leaders agreed that the training would be helpful for advising their Marines about seeking care. That they indicated intent to use the materials with their Marines, also reiterates the perceived value of the toolkit. To a lesser extent, the findings also suggest that the toolkit materials are promising and have the potential to dispel some mental health treatment-related myths associated with seeking help for stress concerns. Training that emphasizes the benefits of getting help and offers opportunities to address real-life experiences and attitudes may reduce stigma associated with seeking help for mental health concerns in a military population, although results from this study are preliminary. Future research is needed to assess if this shift in stigma awareness can be maintained beyond the training. In addition, future studies should investigate the effect of similar yet enhanced interventions based on educational and contact strategies for military personnel to ultimately ensure that all personnel seek appropriate care if needed. Lastly, future research should assess the balance of intervention needed and types of persuasion to shift attitudes specifically for senior enlisted leaders, which will likely be different than their officer counterparts.

## Abbreviations

PTSD: posttraumatic stress disorder
SD: standard deviation
SELs: senior enlisted leaders

## References

[CR1] Acosta JD, Becker A, Cerully JL, Fisher MP, Martin LT, Vardavas R, Slaughter ME, Schell TL (2014). Mental health stigma in the military.

[CR2] Ben-Zeev D, Corrigan PW, Britt TW, Langford L (2012). Stigma of mental illness and service use in the military. J Ment Health.

[CR3] Bray RM, Pemberton MR, Lane ME, Hourani LL, Mattiko MJ, Babeu LA (2010). Substance use and mental health trends among US military active duty personnel: key findings from the 2008 DoD Health Behavior Survey. Mil Med.

[CR4] Brown MC, Creel AH, Engel CC, Herrell RK, Hoge CW (2011). Factors associated with interest in receiving help for mental health problems in combat veterans returning from deployment to Iraq. J Nerv Ment Dis.

[CR5] Corrigan PW, Penn DL (1999). Lessons from social psychology on discrediting psychiatric stigma. Am Psychol.

[CR6] Dickstein BD, Vogt DS, Handa S, Litz BT (2010). Targeting self-stigma in returning military personnel and veterans: a review of intervention strategies. Mil Psychol.

[CR7] Greene-Shortridge TM, Britt TW, Castro CA (2007). The stigma of mental health problems in the military. Mil Med.

[CR8] Hoge CW, Castro CA, Messer SC, McGurk D, Cotting DI, Koffman RL (2004). Combat duty in Iraq and Afghanistan, mental health problems, and barriers to care. N Engl J Med.

[CR9] Jacobson IG, Ryan MAK, Hooper TI, Smith TC, Amoroso PJ, Boyko EJ, Gackstetter GD, Wells TS, Bell NS (2008). Alcohol use and alcohol related problems before and after military combat deployment. J Amer Med Assoc.

[CR10] Joint Mental Health Advisory Team 7 (J-MHAT 7) Operation Enduring Freedom 2010 Afghanistan. Office of the Surgeon General, United States Army Medical Command, Office of the Command Surgeon HQ, USCENTCOM, and Office of the Command Surgeon US Forces Afghanistan (USFOR-A) (2011) http://armymedicine.mil/Pages/reports.aspx

[CR11] Kim PY, Britt TW, Klocko RP, Riviere LA, Adler AB (2011). Stigma, negative attitudes about treatment, and utilization of mental health care among soldiers. Mil Psychol.

[CR12] Milliken CS, Auchterlonie JL, Hoge CW (2007). Longitudinal assessment of mental health problems among active and reserve component soldiers returning from the Iraq War. J Amer Med Assoc.

[CR13] Momen N, Strychacz CP, Viirre E (2012). Perceived stigma and barriers to mental health care in marines attending the combat operational stress control program. Mil Med.

[CR14] Quartana PJ, Wilk JE, Thomas JL, Bray RM, Rae Olmsted KL, Brown JM, Williams J, Kim PY, Clarke-Walper K, Hoge CW (2014). Trends in mental health services utilization and stigma in US Soldiers from 2002 to 2011. Am J Public Health.

[CR15] Reinke RR, Corrigan PW, Leonhard C, Lundin RK, Kubiak M (2004). Examining two aspects of contact on the stigma of mental illness. J Soc Clin Psychol.

[CR16] Sayer NA, Friedemann-Sanchez G, Spoont M, Murdoch M, Parker LE, Chiros C, Rosenheck R (2009). A qualitative study of determinants of PTSD treatment initiation in veterans. Psychiatry.

[CR17] Sharp M, Fear NT, Rona RJ, Wessely S, Greenberg N, Jones N, Goodwin L (2015). Stigma as a barrier to seeking health care among military personnel with mental health problems. Epidemiol Rev.

[CR18] Vogt D (2011). Mental health-related beliefs as a barrier to service use for military personnel and veterans: a review. Psychiatr Serv.

